# Community Health Workers and Stand-Alone or Integrated Case Management of Malaria: A Systematic Literature Review

**DOI:** 10.4269/ajtmh.14-0094

**Published:** 2014-09-03

**Authors:** Lucy Smith Paintain, Barbara Willey, Sarah Kedenge, Alyssa Sharkey, Julia Kim, Valentina Buj, Jayne Webster, David Schellenberg, Ngashi Ngongo

**Affiliations:** Disease Control Department, Faculty of Infectious and Tropical Diseases, and Department of Infectious Disease Epidemiology, Faculty of Epidemiology and Population Health, London School of Hygiene and Tropical Medicine, London, United Kingdom; Malaria Public Health Group, Kenya Medical Research Institute-Wellcome Trust Research Programme, Nairobi, Kenya; United Nations Children's Fund, Health Section, Programme Division, New York, New York

## Abstract

A systematic literature review was conducted to assess the effectiveness of strategies to improve community case management (CCM) of malaria. Forty-three studies were included; most (38) reported indicators of community health worker (CHW) performance, 14 reported on malaria CCM integrated with other child health interventions, 16 reported on health system capacity, and 13 reported on referral. The CHWs are able to provide good quality malaria care, including performing procedures such as rapid diagnostic tests. Appropriate training, clear guidelines, and regular supportive supervision are important facilitating factors. Crucial to sustainable success of CHW programs is strengthening health system capacity to support commodity supply, supervision, and appropriate treatment of referred cases. The little evidence available on referral from community to health facility level suggests that this is an area that needs priority attention. The studies of integrated CCM suggest that additional tasks do not reduce the quality of malaria CCM provided sufficient training and supervision is maintained.

## Introduction

Malaria remains one of the leading causes of preventable mortality worldwide, despite substantial improvements in control over recent years made possible by increased funding. Prompt and effective treatment is a core strategy of national malaria control programs. The Roll Back Malaria Partnership set the target for achieving and maintaining universal coverage meaning that “80% of malaria patients are diagnosed and treated with effective antimalarial medicines, e.g., artemisinin-based combination therapies (ACT), within one day of the onset of illness”.^1^ Since 2010, the World Health Organization has recommended parasitologic diagnosis rather than presumptively treating all fevers as malaria.^2^

Available household survey data on progress towards the goal of universal treatment shows that the median proportion of children less than five years of age with a fever who sought treatment in sub-Saharan Africa during 2010–2011 was 59% (interquartile range [IQR] = 48–76%).^3^ Among children treated with any antimalarial drug, the percentage receiving ACTs varies across countries from < 5% to > 90%.^4^ A crucial factor contributing to low coverage of prompt and effective malaria treatment is the weakness of national health systems to deliver interventions in most low income countries.^5^ One potential solution to improve access is to take services closer to communities by training local community health workers (CHWs) in the provision of diagnostic and treatment services.^6^,^7^ In this report, we define community-based management of malaria as provision of malaria diagnosis and treatment by lay members of the community, without previous formal medical training but trained in basic management of malaria (with or without other basic health services).

A systematic review on the health impact of home-based management of malaria (HMM) was published in 2007 and provided a comprehensive analysis of studies conducted and published in the pre-ACT era.^6^,^8^ More recently, several large-scale studies and programs have been funded that represent a shift in the international focus from HMM or community case management (CCM) of individual diseases to integrated community case management (iCCM).^9^,^10^ This report expands the 2007 review by Hopkins and others^8^ in the current context of use of ACT and rapid diagnostic tests (RDTs) at the community level, which has increased the level of complexity of the tasks for which CHWs are responsible. It is important to understand the impact of interventions on outcomes other than morbidity and mortality,^11^ such as intervention coverage and quality of CHW performance to inform best practice for existing CHW programs and to identify areas where further research may be needed.

We conducted a systematic literature review to assess the evidence for interventions to 1) increase the quality of services provided by CHWs responsible for malaria case management among children less than five years of age; 2) integrate malaria diagnosis and case management with other health services at the community level, with particular emphasis on case management of uncomplicated pneumonia; 3) increase the capacity of health systems to support case management at the community level; and 4) strengthen referrals from the community to facility-based providers.

## Methods

Where relevant, we followed the Preferred Reporting Items for Systematic Reviews and Meta-Analyses statement and checklist in designing and reporting our review.^12^ The online databases CAB Abstracts, EMBASE, Global Health, MEDLINE, and PsychINFO were searched using the terms malaria or fever, community, and home management or case management or treatment or integrated or referral. Gray literature was accessed by searching websites of development agencies and organizations involved in malaria CCM programs. The searches were limited to publications from 2000 onwards, and the last searches were conducted in May 2013. No restriction was placed on language of publication. All records were screened by two reviewers. We restricted inclusion to studies conducted in sub-Saharan Africa, and included randomized controlled trials (RCT), clustered RCTs, pre-post studies with or without control, and interrupted time series designs with at least three time points pre-intervention and post-intervention. Post-only studies were also included where the outcome of interest could be attributed to the community-based intervention under study. For example, through review of CHW registers or household surveys with questions on treatment by source that were conducted after the introduction of the CHW intervention (i.e., before the intervention there was no malaria case management conducted at the community level).

Studies were included if they involved evaluation of an intervention to introduce or improve community-based management of malaria where objective and standardized impact or outcome measures were reported. If linked to an included publication describing a community-based treatment intervention, qualitative studies, case studies, process evaluations, and cost-effectiveness studies were included to identify barriers to and facilitators of effective implementation.

Studies with an RCT or controlled pre-post design were assessed by two authors for risk of bias, based on guidelines from the Cochrane Effective Practice and Organization of Care group^13^; each study was given a classification of DONE, NOT CLEAR, or NOT DONE for the categories sample size calculations, randomization and blinding procedures (where appropriate), similarity of intervention and control areas at baseline, and completion of participant follow-up.

Because of heterogeneity between studies in terms of the intervention and study design, it was not appropriate to conduct a meta-analysis. Instead, content analysis and narrative synthesis were used to summarize findings and identify important influences on the quality of malaria CCM across studies.^14^

## Results

**Figure 1.**
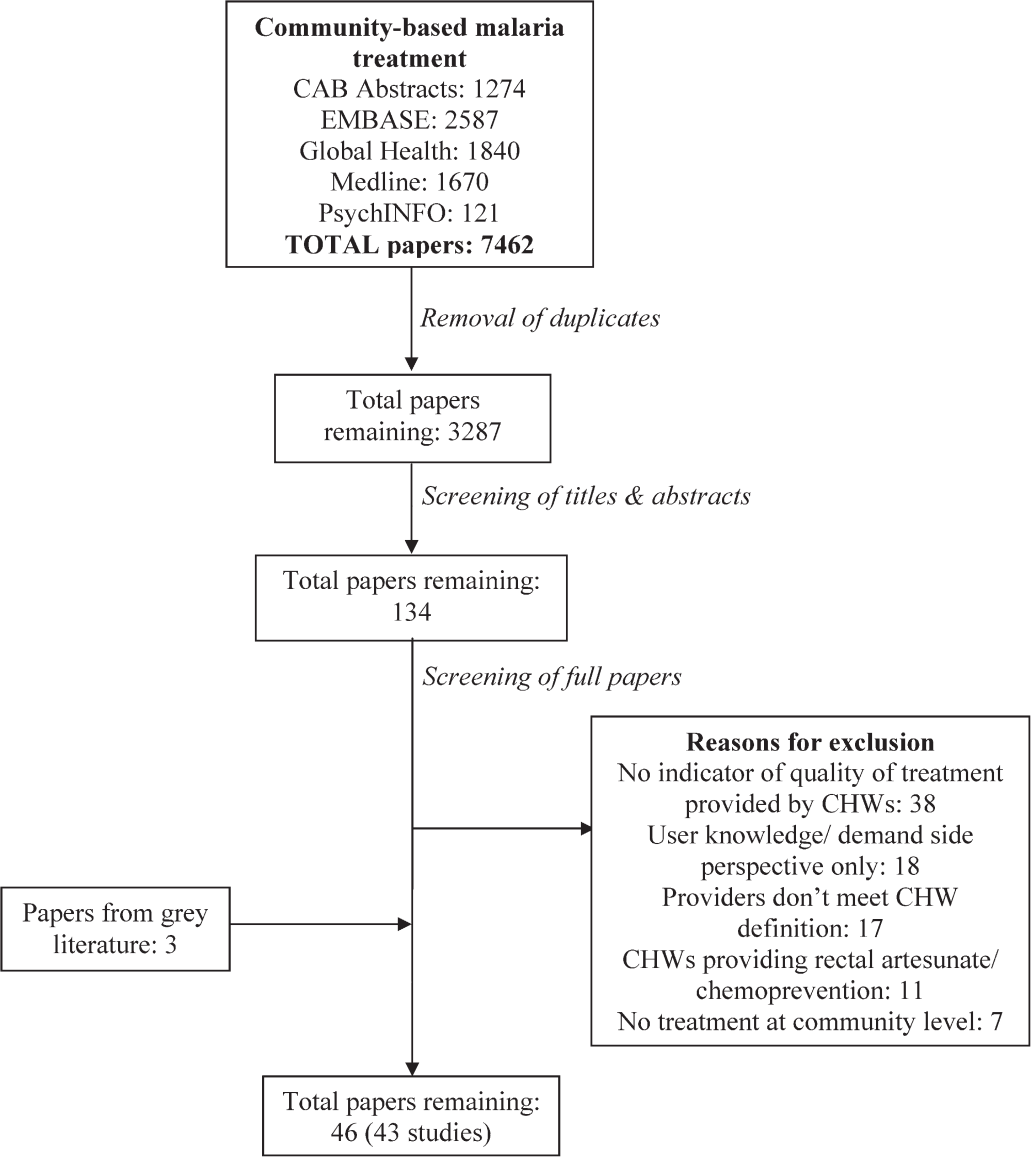
Flow chart of literature search and screening. CHWs = community health workers.

A total of 3287 papers were found by searching the online publications databases. After screening, 43 papers from the published literature remained for final analysis. An additional three papers were included from the gray literature (Figure 1). The final 46 papers cover a total of 43 studies, where an individual study is defined as an evaluation of an intervention conducted in one site (country). The final 43 studies were conducted in 16 countries; 25 studies in eastern or southern Africa and 18 in western or central Africa. Most (81.4%, 35 of 43) studies were conducted in rural settings.

### Quality of included studies.

Twelve studies used a cluster RCT to evaluate the intervention,^15^–^23^ three used a controlled pre-post design,^24^–^26^ one pre-post without control,^27^ and five studies were post-only with a control.^28^–^30^ All of the 22 post-only studies without a control involved a direct evaluation of CHW performance (e.g., by review of CHW registers) or household surveys with questions on treatment by source; thus, the quality of performance could be related to the intervention and any contextual factors described.^31^–^43^ Although the post-only studies did not have a baseline comparison, the evaluations were conducted after the introduction of the CHW intervention (i.e., before the intervention, there was no previous malaria case management conducted at the community level). Thus, the quality of CHW performance was directly ascribed to the intervention package that they experienced.

Overall, the quality of the 12 cluster RCTs was reasonably high, particularly in appropriateness and clarity of sample size calculations and minimization of selection bias through randomization. Similarity of the intervention and control arms at baseline was generally good in terms of the primary outcome of interest and general socio-demographic indicators, and the quality of studies in terms of measuring and reporting completion of follow-up for study participants was mixed (Table 1 ).

The quality of the three studies with a controlled pre-post design was considerably lower than the RCTs: All three studies found significant differences at baseline in primary outcome and socio-demographic characteristics between the intervention and control groups, which makes interpretation of results difficult because it is not clear whether any changes in outcome are caused by the intervention or to contextual factors (Table 1).

**Figure 2.**
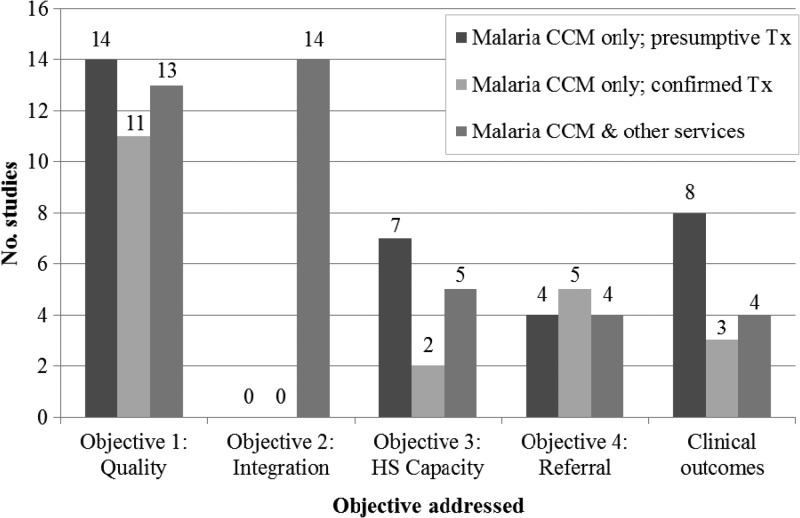
Number of studies addressing each objective, by study design. RCT = randomized controlled trial; HS = health system.

The RCTs provided the greatest amount of evidence for interventions that integrated other health services with malaria CCM and those that measured clinical outcomes (Figure 2). The evidence for interventions to improve quality of CHW performance, health system capacity, and referrals was drawn primarily from post-only studies without control groups. However, RCTs provided the next most common evidence source. Clinical and non-clinical performance-related outcomes were measured by both types of study and results were not noticeably different in magnitude or direction according to the quality of the study design.

### Characteristics of the interventions.

**Figure 3.**
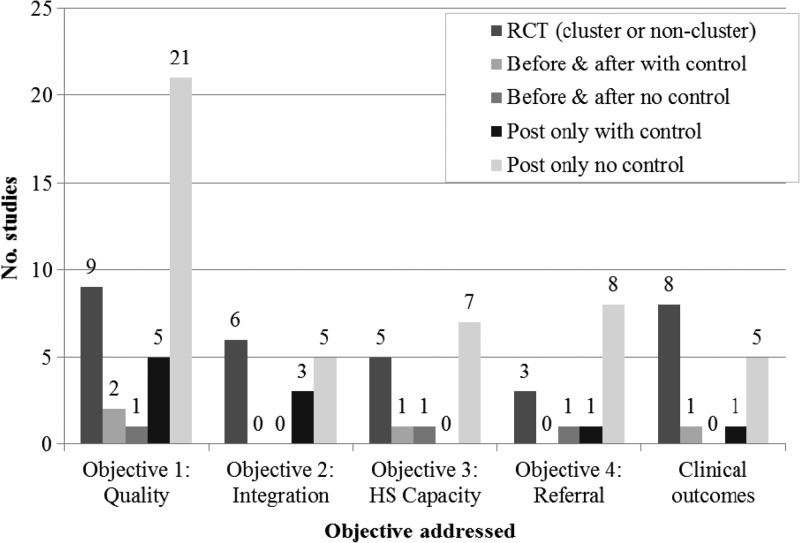
Number of studies addressing each objective, by classification of intervention. CCM = community case management; Tx = treatment; HS = health system.

Twenty-nine studies evaluated malaria CCM alone, 16 of these by presumptive diagnosis and 13 by confirmed diagnosis; the other 14 studies evaluated malaria CCM integrated with other basic health services (Supplemental Table 2). Thirty-eight studies reported on indicators of quality of case management by CHWs, (objective one of this review); 14 investigated integration of malaria CCM with other health services (objective two)^20^–^23^,^28^,^32^,^39^,^41^,^44^–^46^; 14 reported on some element of health system capacity to support malaria CCM, such as supervision, supply chain management, or treatment of referred cases (objective three)^15^,^17^,^23^,^24^,^27^,^33^,^35^,^38^,^42^,^43^,^46^; 13 provided information on referrals from the community to health facility level (objective 4),^15^,^18^,^27^,^30^,^33^–^35^,^38^,^39^,^41^,^43^,^45^,^46^ and 15 studies reported clinical outcomes^15^–^17^,^19^,^21^,^23^,^25^,^30^,^40^,^42^,^47^ (Figure 3).

The profiles of the CHW programs varied in terms of whether there were any incentives for the CHW or user charges. The CHWs in 14 of the studies received some financial incentive, ranging from a small commission of a few US cents per dose of antimalarial drug sold,^17^,^31^,^32^,^34^,^35^,^42^ to a monthly allowance of US$15 or US$25 in a couple of cases.^18^,^32^,^40^,^46^ In eight studies, non-financial incentives were provided in the form of bicycles or other equipment or payment in kind from neighbours^20^,^27^,^28^,^30^,^31^,^33^; in four, intrinsic motivation such as pride and satisfaction in serving their community was specified.^20^,^28^,^35^,^46^ Only four studies explicitly stated that no motivation was given,^19^,^21^,^22^,^26^ although 17 studies did not provide any information on CHW motivation. Of the 43 studies, 12 charged the user a small amount per dose of antimalarial drug (ranging from US$0·05 for chloroquine to US$0·45 for an ACT).^30^,^35^ In 18 studies, antimalarial drugs were distributed free;^15^,^18^–^20^,^24^,^26^–^29^,^31^–^33^,^38^,^40^,^43^,^44^,^46^ and the remaining 13 studies did not provide information on charges to the user. The size of population covered by each CHW also varied (median = 692 persons per CHW; IQR = 409–1,740), as did the sex of CHWs (median = 43% male; IQR = 25–72%). However, certain characteristics were consistent across the studies, including that: CHWs were members of the community that they served and for the most part were elected during public village meetings; and minimum requirements for a CHW were basic literacy, availability and accessibility, and a willingness to volunteer and serve.

### Interventions to improve quality of CHW performance: malaria CCM alone.

Results from the 16 studies that involved presumptive treatment of malaria (without integration with other basic health services) found that CHW performance was high in terms of delivering the correct antimalarial drug and at the correct dose; among the nine studies that reported this outcome indicator, the median proportion was 97.8% (IQR = 94.8–98.0%).^15^,^27^,^31^,^32^,^35^ Prompt treatment seeking from a CHW was also high at approximately 90%, although this factor was only reported for four of the studies of presumptive treatment of malaria.^31^ An additional four studies reported on a combined indicator of prompt treatment with the correct dose, which was lower (median = 69.8%; IQR = 55.2–75.6%).^19^,^31^,^32^,^42^ Taking into consideration changes in the comparison areas, both studies with a controlled pre-post design found an overall increase in prompt and effective treatment that could be ascribed to CHWs (47.6% in Tanzania and 13.5% in Uganda).^17^,^48^

The evaluation of the national HMM program in Uganda and a regional program in Burkina Faso are the largest studies in the review and were conducted with limited external support for supervision or antimalarial drug supply. These programs showed more modest achievements in terms of CHW performance: 32.3% of children treated with pre-packaged chloroquine–sulfadoxine/pyrimethamine in Uganda received the correct dose within 24 hours^48^; in Burkina Faso, 56.4% of febrile children were treated promptly with a pre-packaged antimalarial drug.^42^ Reasons offered by the authors include the need for more community mobilization and stronger supervision in Uganda, and a need for improvements to the pre-packaging in Burkina Faso, which was conducted before blister packs were commonly available.^42^,^48^

The 13 studies that investigated RDT-confirmed diagnosis and treatment of malaria showed encouraging results in the ability of CHWs to use RDTs and treat according to the result when they received practical training with an opportunity for observation of practice and problem solving. Three of the studies that measured the quality of CHW performance according to the number of steps correctly conducted in preparing and conducting an RDT, and the proportion that read the result correctly^29^,^36^,^49^ reported substantial and statistically significant improvements (Supplemental Table 2). Appropriate treatment according to RDT result in terms of proportion of RDT-positive and RDT-negative patients treated with ACT was reported by seven and four studies, respectively. Among these studies, appropriate treatment by the CHWs was high (median = 97.7% RDT-positive patients receiving ACT; IQR = 92.7–99.3%^18^,^30^,^32^–^34^,^37^ and median = 8.4% of RDT-negative patients receiving ACT; IQR = 4.6–15.5%).^18^,^33^,^34^,^37^

### Interventions to improve quality of CHW performance: integration of malaria CCM with other health interventions.

The findings from 14 studies in which CHWs delivered other child health services in addition to malaria CCM were consistent with the malaria-only programs in terms of achieving high adherence to malaria CCM algorithms, both presumptive and confirmed (Supplemental Table 2). Two notable exceptions were the community-directed intervention study in Cameroon, in which only 27.9% (19 of 68) of febrile children received prompt and effective treatment after two years of intervention,^28^ and a study in Kenya, in which 41.0% (39 of 95) of febrile children received the correct dose of antimalarial drug after 12 months of intervention.^39^ In Cameroon, national policy changed from presumptive to confirmed diagnosis during the intervention, which meant that malaria CCM could not continue. In Kenya, refresher training after the first evaluation led to improvements with 92.7% (115 of 124) and 90.5% (237 of 262) of febrile children receiving the correct dose of antimalarial drug after refresher training after two and four years of intervention, respectively.

The CHWs in nine of the integrated studies were trained to diagnose uncomplicated pneumonia and treat with antibiotics. A median of 75.8% (IQR = 60.0–81.0%) of children given a diagnosis of pneumonia received an antibiotic among eight studies that reported the outcome.^20^,^22^,^23^,^39^,^41^,^46^ However, the proportion of pneumonia diagnoses made by CHWs that agreed with a clinician gold standard was lower and variable (median = 63.7%; IQR = 47.1–77.7%) among the six studies that reported this outcome.^22^,^39^,^41^,^44^,^46^

For example, problems measuring respiratory rates and classifying pneumonia were reported by Gilroy and others in their evaluation of the iCCM program in Malawi in which only 51.7% (30 of 58) of children with fast breathing and cough were correctly classified, of whom 83.3% (25 of 30) received an antibiotic.^46^ Conversely, in an evaluation of CHW performance conducted immediately after training on the use of RDT for malaria diagnosis and respiratory timers for pneumonia diagnosis in Uganda, 84.6% (154 of 182) of respiratory timer readings were in agreement with those of the study clinician.^44^ Likewise, in a cluster RCT in Zambia, CHWs followed the classification algorithm well, classifying 98.3% (362 of 378) of children with fast breathing as having pneumonia.^50^ The positive results observed by Mukanga and others in Uganda immediately after training^44^ were maintained over the 12-month follow-up period of the cluster RCT; 98.3% (520 of 529) of children with a high respiratory rate in the intervention arm received an antibiotic compared with only 0.90% (4 of 446) of children with a normal breathing rate.^23^ Results from Burkina Faso and Ghana, the two other countries involved in this multi-site study, were not quite as impressive: 79.0% (181 of 229) and 72.5% (103 of 142) of children with high respiratory rate were given antibiotics, respectively. Overuse of antibiotics was of greater concern; 38.5% (114 of 296) of children with a normal respiratory rate received antibiotics in Burkina Faso and 44.6% (197 of 442) of children with a normal respiratory rate received antibiotics in Ghana.^23^ The authors suggested that more intensive supervision in Uganda may explain the difference between countries.

### Interventions to strengthen health system support for CCM.

There were few studies that specified a separate health worker or health system strengthening component to the malaria CCM intervention. Eleven studies reported specifically on training health workers to support CHWs; e.g., retraining health workers on malaria case management,^15^,^17^,^20^,^23^ orientating health workers to accept and treat referrals sent by CHWs,^23^,^27^,^43^,^45^ or training on how to supervise the CHWs and collect reporting forms and deliver drug supplies;^23^,^35^,^50^ the impact of this training was not clear from the papers. Two studies reported that pharmacists were trained to strengthen stock management at the health facility and maintain the supply chain to the community level; good availability of antimalarial drug stock was reported for both studies during the evaluation.^35^,^42^ However, few other studies reported any indication of continuity of antimalarial drug stock during the intervention.

Supervision was generally described as part of the context in which the CHWs operated, rather than as the focus of the intervention itself. Frequency of supervision varied between studies, and the most commonly reported frequency was monthly (although it was not always clear whether this was the intended or actual frequency of supervision). In general, those CHWs supervised by a research team were visited more often than those visited by public health workers; in some cases this finding was as frequent as two or three times a week. For studies in which the study team was involved in supervision, regular and frequent supervisory visits by experienced personnel were found to be a facilitator of good CHW performance.^15^,^17^,^20^,^23^,^27^,^31^–^33^,^37^

Among the studies that reported problems with supervision, most related to health system weaknesses, such as staff shortages and competing priorities,^28^,^35^,^41^,^43^ insufficient skills,^24^,^39^ or a lack of fuel for transportation.^24^,^28^ Two reports provided illustrations of how poor supervision can reduce community and CHW confidence, which could potentially undermine the malaria CCM intervention: negative experiences of CHWs with formal health workers in Nigeria led them to bypass health facilities in favor of supervision by community leaders and provision of drug supplies directly from the district stores^51^; and early evaluation of the HMM program in Uganda found that CHWs and communities were concerned about lack of supervisory support from health workers, which affected motivation.^48^

Interestingly, the qualitative data was supported by higher levels of CHW performance in those studies in which supervision was discussed in a positive light than in those studies in which there were negative comments by the CHWs or communities: for 13 of the positive supervision studies with a common outcome indicator, the median proportion of children with malaria that were treated appropriately was 97.8% (IQR = 94.0–99.5%). Conversely, for 10 of the negative supervision studies with a common outcome indicator, the median proportion was 78.0% (IQR = 45.4–89.1%). Although the median was still reasonably high, the range of results was broader, indicating that the quality of study outcome was more variable.

### Interventions to strengthen referrals from community to facility-based providers.

Less than one-third (13) of the studies reported an indicator of referral, either the proportion of patients referred to a health facility^15^,^18^,^27^,^30^,^34^,^35^,^38^,^39^,^41^,^43^,^45^,^46^ and/or the proportion that completed their referral (i.e., those who attended a health facility after being referred there by a CHW).^27^,^33^,^35^,^38^,^43^,^45^ For the eight studies that reported the proportion of all patients referred, the median referral rate was 17.0% (IQR = 8.0–18.8%)^15^,^18^,^34^,^38^,^41^,^43^,^45^; the most common reasons for referral (where reported) were signs of severe disease or symptoms that the CHW did not have the capacity to treat. It is not clear whether all cases that should have been referred were referred. Three studies specifically reported the proportion of severe cases that were correctly referred, ranging from 29.4% to 70.0%.^27^,^39^,^46^ Median completion of referrals among the six studies that reported the indicator was 67.1% (IQR = 55.1–86.0%).^27^,^33^,^35^,^38^,^43^,^45^

Only two of the studies specifically evaluated interventions to improve the referral system from the community to formal health facility level. The first study in Sierra Leone reported a higher completion rate (approximately 90%) for patients with severe malaria who were referred to a health center than for RDT-negative patients (only 1%) after introduction of new referral registers with training at the community and facility levels.^43^ The second study in Mali reported increases in the proportion of children referred and completing referral after an intervention to strengthen the referral system and two-way communication between the CHW and health staff, although there was a discrepancy between the results of a household survey and the CHW referral record review. After further investigation, the authors reported that CHWs were more likely to give informal verbal referral advice to those patients whom they did not expect to complete the referral, but make a formal written reference for those patients who were likely to comply.^45^

Studies in Uganda and Kenya also found evidence of CHWs making judgments on which patients to refer, reporting that they were cautious about referring patients unnecessarily and causing them to incur costs that they could not afford,^35^ often choosing instead to monitor patients at home rather than refer immediately.^39^ Another study in Uganda reported that urgent cases, children less than one year of age, and those to whom clear instructions were given, were more likely to complete the referral.^38^

### Impact of malaria CCM intervention on clinical outcomes.

Eight of the 16 studies of presumptive treatment of malaria and 3 of the 13 studies involving diagnosis with RDTs reported clinical outcomes; of these 11 studies, only four were cluster RCTs. The significant reductions in all-cause mortality and severe malaria morbidity, respectively, shown by an RCT study in Ethiopia and a case–control study in Burkina Faso in the early 2000s provided strong support for the early home-based treatment of fever programs.^16^,^42^ A more recent study in Ethiopia also reported a significant reduction in malaria-attributable deaths in the intervention district in which CHWs had RDTs and ACTs, compared with a control district without malaria CCM; no significant difference in all-cause mortality was observed.^25^ In Senegal, total deaths and deaths attributed to malaria showed a greater decrease in regions with malaria CCM compared with those that had not yet rolled out the intervention. However, there was no significant decrease in outpatient malaria cases or hospitalizations between intervention and comparison regions.^30^ Similarly, RCTs of home management of malaria in Uganda, Burkina Faso, and Tanzania did not find a significant impact on morbidity outcomes such as moderate anemia, hemoglobin level, or splenomegaly.^15^,^17^,^19^ One encouraging finding in terms of clinical outcomes is that among four studies that measured day 28 polymerase chain reaction–adjusted cure rates, > 90% of patients had completely cleared their infection after treatment with an ACT from a CHW, supporting the idea that CHWs can prescribe the appropriate dose and that users adhere to this dose.^40^,^47^

Four of the 14 integrated studies reported clinical outcomes (all of which were cluster RCTs). A three-arm cluster RCT in Ghana reported a 44% reduction in all-cause child mortality among febrile children treated presumptively with an ACT and antibiotic compared with standard care (adjusted relative risk [RR] = 0.56, 95% confidence interval [CI] = 0.41–0.76, *P* < 0.001). Presumptive treatment with an ACT alone also significantly reduced child mortality by 30% compared with standard care (adjusted RR = 0.70, 95% CI = 0.53–0.92, *P* = 0.01). However, there was no significant difference between the two intervention arms (RR = 0.79, 95% CI = 0.56–1.12, *P* = 0.20).^21^ The multi-site cluster RCT in Burkina Faso, Ghana, and Uganda reported high fever clearance (> 95%) across all three countries. However, there were no significant differences between the intervention arms (treatment with ACTs and antibiotics according to diagnoses made with RDTs and respiratory timers) and control arms (presumptive treatment with ACTs).^23^

## Discussion

Overall, the quality of malaria case management at the community level was high across most studies included in this review. In particular, extremely high compliance by CHWs to the correct dose was seen across the studies that reported this outcome; this finding was irrespective of diagnosis or antimalarial drug policy, or strength of study design. All except one of the studies reporting this indicator involved pre-packaged antimalarial drugs (co-blistered or co-formulated).^45^ Although direct data on user adherence to the correct dose was not generally reported, four studies investigated polymerase chain reaction–adjusted cure rates of a sub-sample of patients treated with ACT by a CHW, all of which were > 90%.^40^,^47^ This finding supports the notion that pre-packaged ACTs can be administered effectively by CHWs and adhered to by the users of these services.

Appropriate treatment at the community level according to RDT result was higher than that found in a number of studies of RDT use by formal health workers, in which 30–80% of RDT-negative patients were treated with antimalarial drugs.^52^–^56^ Reasons for this lack of adherence are varied and complex, involving trust in RDTs, maintaining clinical reputation, managing patient expectations, and availability of alternative treatments.^56^–^58^

In contrast to the positive findings on quality of malaria CCM, the evidence from this review suggests a more mixed performance for community-level management of pneumonia in the context of integration with malaria diagnosis and treatment. In general, the greatest challenge seems to be in measuring respiratory rate and/or classifying pneumonia; treatment with an antibiotic of patients classified as having pneumonia (rightly or wrongly) is reasonably high, provided sufficient stock is available.^23^ Studies in which pneumonia classification was in close agreement with the study clinician gold standard were cluster RCTs with intensive supervision and follow-up.^20^,^44^,^50^ As Kalyango and others reported, it is possible that in many settings with an established malaria CCM program the CHWs are familiar with the signs, symptoms, and treatment of malaria, whereas legal permission to treat pneumonia at the community level is a more recent addition to their responsibilities.^22^ The quality of CCM of pneumonia may improve with continued support, such as supervision and/or refresher training with opportunity for problem solving.

Less than one-third of the studies included in this review reported on referrals between the community and health facility levels. Notwithstanding that indicators can be difficult to monitor, this is a low level of reporting considering that effective referral of cases that a CHW does not have the capacity to treat is a crucial element of safe implementation of CCM. Evidence from included studies suggests that CHWs and patients make judgment decisions around the referral process. When CHWs were trained to treat all cases of fever as malaria, criteria for referral were relatively straightforward, generally involving training on recognition of danger signs and referral of such patients to a health facility after treatment with an antimalarial drug. However, with the use of RDTs by CHWs, there arises a challenge of what action should be taken for patients with a negative RDT result. In the longer term, development of additional point-of-care diagnostic tests to complement malaria RDTs and to help differential diagnosis is likely to be helpful. In the meantime, provision of clear guidance on appropriate management of undiagnosed fevers and clear process monitoring of CHW referral systems and qualitative studies of provider and caretaker behavior around referrals are essential to ensure patient safety.

Supply chain strengthening, including forecasting and monitoring, is vital in ensuring that CHWs can effectively carry out their task. Likewise, capacity of health workers at primary public health facilities in the integrated management of childhood illnesses and management of severe malaria or pneumonia is an important factor to the overall success of community case management because under routine conditions, it is these health workers who have the responsibility for supervising the practice of CHWs and treating referred cases. Few of the studies included in this review reported quantitative indicators of health worker capacity, and despite the likely need, only approximately 25% of studies specified any health worker training as part of the intervention. With the exception of two studies that successfully improved CHW stock continuity through training of health facility pharmacists,^35^,^42^ it was not always clear to what extent such intervention had been effective. A recent systematic review and meta-analysis concluded that training in integrated management of childhood illnesses could improve quality of treatment of childhood illnesses at health facility level, particularly when implemented in conjunction with greater supervision.^59^ The variety of challenges for those studies in this review that did not have intensive involvement of a research team also support that training alone is insufficient to improve health system capacity; more in-depth assessment of the needs at the supervising health facility level will be needed to design appropriate interventions to support CCM.

Sufficient supportive supervision is a recurrent facilitating factor in studies of health worker performance at all levels of the health system, and this review reinforces its importance in maintaining the quality of CCM. In general, supervision was more frequent in the studies conducted under controlled research conditions compared with studies that evaluated larger scale real-life implementation of malaria CCM. Although it is not possible to make quantitative conclusions because of differences in intervention details and study design, qualitative analysis suggests that when supervision is considered by CHWs to be sufficiently frequent and supportive, this factor can encourage higher CHW motivation and performance. In addition, respect and support from formal health workers can build trust of CCM within the community. This finding is supported by another recent literature review, which suggested that to understand treatment seeking behavior fully, characteristics of providers^60^ should be considered in addition to those of users.^61^ Kizito and others reported that traditional barriers such as cost and proximity are important; however, they also report that health services may increase their appeal if they responded to user preferences for friendliness, effectiveness, compassion, and shorter waiting times.^60^ Further research is needed to translate effective supervision strategies from research settings to scaled-up programs.

It is not possible to comment on the achievement of one of the fundamental objectives and stated strengths of CHWs in improving socioeconomic (and geographic) access to care^6^ because it was rarely reported. Often CHW programs are designed to target hard-to-reach communities (e.g., those > 5 km from a health facility or in the lowest socioeconomic quintiles). It may be that equity was incorporated into the design of the intervention rather than being directly measured. Nevertheless, whenever possible, it is important to evaluate whether a CHW program has actually achieved improved equity of access to included interventions.

Another area of limited evidence is cost-effectiveness of malaria CCM. Nine of the included studies reported a costing element,^19^,^28^–^30^,^36^,^62^,^63^ with variations in purpose and method of data collection. Although malaria CCM appears to be a less costly approach compared with standard care for malaria treatment at a health facility, the variety of costing methods means it is not possible to present a clear picture of the cost-effectiveness of the approach. In particular, data are needed for indirect opportunity costs for CHWs volunteering their time.^64^ More economic evaluations of malaria CCM are needed, and standardization of methods to enable cross-study comparison will be extremely valuable.

As this and other recent reviews highlight, there is still relatively little evidence that CHWs can impact on mortality or morbidity,^11^,^65^ although there is reasonable evidence they can perform well with adequate supervision and health system support. Health impact studies rarely reported process or outcome indicators that would help understand health outcomes and relate these to the strength of implementation.^16^,^25^ Similarly, most of the program evaluations focused on process and outcome indicators but did not report on health outcomes (and were not designed to do so). This finding suggests an area for further investigation, namely for studies that are powered to measure impact of malaria CCM on morbidity or mortality to also include process evaluation in their design so that inference can be drawn on the level of implementation of malaria CCM needed to achieve health impact.

It is important to note the potential limitations in terms of the reliability of data extracted from the included studies. Many of the studies reported data on antimalarial drug use and dosage from reviews of the CHW registers, which may have incomplete or inaccurate reporting. Others report the proportion of febrile children receiving an effective antimalarial drug at the correct dose from household surveys, which can have problems with recall and understanding. Direct observation of CHW performance is considered the gold standard, although this observation has implications for influencing behavior and is time and resource intensive and thus is not suitable for routine monitoring. However, despite these limitations, the small number of studies that collected data from CHW registers and household surveys to triangulate data generally found encouragingly similar results.^31^,^32^ Where possible, validation of data from different sources is desirable.

In conclusion, the findings from this review support the potential for CHWs to implement quality care of malaria at the community level, even with increasing complexity of their roles and responsibilities. More evidence is emerging on the integration of malaria CCM with other health services, particularly from programs that follow iCCM approach. CHWs are able to treat uncomplicated pneumonia, although there is room for improvement, particularly in accurate diagnosis. Important facilitating factors for success include interactive and practical training, clear guidelines, and regular supportive supervision. However, there is an urgent need for more evidence on supervision in the context of scaled programs, as well as the most appropriate course of action for RDT-negative patients and the effective referral by CHWs of cases that they are unable to treat. It is a cause for concern that such limited evidence exists on this area, given the crucial role that referral must play in ensuring patient safety when responsibility for case management is transferred to low-level community providers.

## Supplementary Material

Supplemental Tables.

## Figures and Tables

**Table 1:** Summary of study quality for randomised controlled trials and controlled pre-post studies included in the review*

Study	Power calculation	Concealment of allocation	Completeness of follow-up	Blinding of primary outcome	Similarity of intervention and control groups at baseline: primary outcome	Similarity of intervention and control groups at baseline: demographics
Randomized controlled trials						
Staedke and others, 2009^19^	Done	Done	Done	Not clear	Done	Done
Mubi and others, 2011^18^	Done	Done	Done	Not clear (not specified)	Done	Done
Chinbuah and others, 2012^21^	Done	Done	Done	Done	Done	Not done (significant difference in ITN use and parasitemia)
Winch and others, 2003^45^	Done	Done	Not clear (no trial profile)	Not clear	Not clear (not reported)	Done
Yeboah-Antwi and others, 2010^20^	Done	Done	Done	Not clear	Not clear (not reported)	Not done (signification difference in immunization)
Mukanga and others, 2012†^23^	Done	Done	Done	Not done	Done	Done
Kidane and Morrow, 2000^16^	Done	Done	Not done	Not clear (not specified)	Done	Not clear (not reported)
Eriksen and others, 2010^15^	Done	Done	Not clear (no trial profile)	Not done	Not done (significant difference in hemoglobin level and parasite positivity)	Done
Kouyate and others, 2008^17^	Done	Done	Not clear (no trial profile)	Not done	Done	Not done (significant difference in ethnicity)
Kalyango and others, 2012^22^,^66^,^67^	Not clear (not reported)	Done	Not clear (no trial profile)	Not clear (not specified)	Not clear (not reported)	Not clear (not reported)
Controlled pre-post studies						
Fapohunda and others, 2004^24^	Done	NA	Not clear (not reported)	Not clear	Not clear (significant difference in prompt treatment seeking and anemia)	Not clear (not reported)
Nsungwa-Sabiiti and others, 2007^26^	Done	NA	Not done	Not done	Not clear (similar on overall treatment, different treatment < 24 hours)	Not clear (descriptive text, no clear table)
Lemma and others, 2010^25^	Done	NA	Not done	Not done	Not done (significant difference in parasite positivity)	Not done (significant difference in altitude and distance to HF)

* ITN = insecticide-treated net; NA = not applicable; HF = health facility.

† Multi-site study (Burkina Faso, Ghana, Uganda) with standardized study design.

## References

[1] Roll Back Malaria (2005). Global Strategic Plan: Roll Back Malaria 2005–2015.

[2] World Health Organization (2010). Guidelines for the Treatment of Malaria.

[3] World Health Organization (2012). World Malaria Report 2012.

[4] United Nations Childrens Fund (2013). World Malaria Day 2013. Invest in the Future: Defeat Malaria.

[5] World Health Organization (2007). Everybody's Business: Strengthening Health Systems to Improve Health Outcomes: WHO's Framework for Action.

[6] World Health Organization (2005). The Roll Back Malaria Strategy for Improving Access to Treatment through Home Management for Malaria.

[7] Haines A, Sanders D, Lehmann U, Rowe AK, Lawn JE, Jan S, Walker DG, Bhutta Z (2007). Achieving child survival goals: potential contribution of community health workers. Lancet.

[8] Hopkins H, Talisuna A, Whitty CJ, Staedke SG (2007). Impact of home-based management of malaria on health outcomes in Africa: a systematic review of the evidence. Malar J.

[9] World Health Organization/United Nations Childrens Fund (2012). WHO/UNICEF Joint Statement: Integrated Community Case Management (iCCM): An Equity-Focused Strategy to Improve Access to Essential Treatment Services for Children.

[10] de Sousa A, Tiedje KE, Recht J, Bjelic I, Hamer DH (2012). Community case management of childhood illnesses: policy and implementation in Countdown to 2015 countries. Bull World Health Organ.

[11] Christopher JB, Le May A, Lewin S, Ross DA (2011). Thirty years after Alma-Ata: a systematic review of the impact of community health workers delivering curative interventions against malaria, pneumonia and diarrhoea on child mortality and morbidity in sub-Saharan Africa. Hum Resour Health.

[12] Moher D, Liberati A, Tetzlaff J, Altman DG, PRISMA Group (2009). Preferred reporting items for systematic reviews and meta-analyses: the PRISMA statement. BMJ.

[13] Cochrane EPOC (2010). Cochrane Effective Practice and Organisation of Care (EPOC) Group: Resources for Review Authors.

[14] Popay J, Roberts H, Sowden A, Petticrew M, Arai L, Rodgers M, Britten N, Roen K, Duffy S (2006). Guidance on the conduct of narrative synthesis in systematic reviews: a product of the ESRC methods programme.

[15] Eriksen J, Mujinja P, Warsame M, Nsimba S, Kouyate B, Gustafsson LL, Jahn A, Muller O, Sauerborn R, Tomson G (2010). Effectiveness of a community intervention on malaria in rural Tanzania - a randomised controlled trial. Afr Health Sci.

[16] Kidane G, Morrow RH (2000). Teaching mothers to provide home treatment of malaria in Tigray, Ethiopia: a randomised trial. Lancet.

[17] Kouyate B, Some F, Jahn A, Coulibaly B, Eriksen J, Sauerborn R, Gustafsson L, Tomson G, Becher H, Mueller O (2008). Process and effects of a community intervention on malaria in rural Burkina Faso: randomized controlled trial. Malar J.

[18] Mubi M, Janson A, Warsame M, Martensson A, Kallander K, Petzold MG, Ngasala B, Maganga G, Gustafsson LL, Massele A, Tomson G, Premji Z, Bjorkman A (2011). Malaria rapid testing by community health workers is effective and safe for targeting malaria treatment: randomised cross-over trial in Tanzania. PLoS ONE.

[19] Staedke SG, Mwebaza N, Kamya MR, Clark TD, Dorsey G, Rosenthal PJ, Whitty CJ (2009). Home management of malaria with artemether-lumefantrine compared with standard care in urban Ugandan children: a randomised controlled trial. Lancet.

[20] Yeboah-Antwi K, Pilingana P, Macleod WB, Semrau K, Siazeele K, Kalesha P, Hamainza B, Seidenberg P, Mazimba A, Sabin L, Kamholz K, Thea DM, Hamer DH (2010). Community case management of fever due to malaria and pneumonia in children under five in Zambia: a cluster randomized controlled trial. PLoS Med.

[21] Chinbuah MA, Kager PA, Abbey M, Gyapong M, Awini E, Nonvignon J, Adjuik M, Aikins M, Pagnoni F, Gyapong JO (2012). Impact of community management of fever (using antimalarials with or without antibiotics) on childhood mortality: a cluster-randomized controlled trial in Ghana [Special supplement on Integrated Community Case Management]. Am J Trop Med Hyg.

[22] Kalyango JN, Rutebemberwa E, Alfven T, Ssali S, Peterson S, Karamagi C (2012). Performance of community health workers under integrated community case management of childhood illnesses in eastern Uganda. Malar J.

[23] Mukanga D, Tiono AB, Anyorigiya T, Kallander K, Konate AT, Oduro AR, Tibenderana JK, Amenga-Etego L, Sirima SB, Cousens S, Barnish G, Pagnoni F (2012). Integrated community case management of fever in children under five using rapid diagnostic tests and respiratory rate counting: a multi-country cluster randomized trial [Special supplement on Integrated Community Case Management]. Am J Trop Med Hyg.

[24] Fapohunda B, Plowman B, Azairwe R, Bisorbowa G, Langi P, Kato F, Wang X (2004). Home-Based Management of Fever Strategy in Uganda: A Report of the 2003 Survey.

[25] Lemma H, Byass P, Desta A, Bosman A, Costanzo G, Toma L, Fottrell E, Marrast A-C, Ambachew Y, Getachew A, Mulure N, Morrone A, Bianchi A, Barnabas GA (2010). Deploying artemether-lumefantrine with rapid testing in Ethiopian communities: impact on malaria morbidity, mortality and healthcare resources. Trop Med Int Health.

[26] Nsungwa-Sabiiti J, Peterson S, Pariyo G, Ogwal-Okeng J, Petzold MG, Tomson G (2007). Home-based management of fever and malaria treatment practices in Uganda. Trans R Soc Trop Med Hyg.

[27] Chinbuah AM, Gyapong JO, Pagnoni F, Wellington EK, Gyapong M (2006). Feasibility and acceptability of the use of artemether-lumefantrine in the home management of uncomplicated malaria in children 6–59 months old in Ghana. Trop Med Int Health.

[28] CDI Study Group (2008). Community-Directed Interventions for Major Health Problems in Africa: A Multi-Country Study: Final Report.

[29] Harvey SA, Jennings L, Chinyama M, Masaninga F, Mulholland K, Bell DR (2008). Improving community health worker use of malaria rapid diagnostic tests in Zambia: package instructions, job aid and job aid-plus-training. Malar J.

[30] Thiam S, Thwing J, Diallo I, Fall FB, Diouf MB, Perry R, Diop M, Diouf ML, Cisse MM, Diaw MM, Thior M (2012). Scale-up of home-based management of malaria based on rapid diagnostic tests and artemisininbased combination therapy in a resource-poor country: results in Senegal. Malar J.

[31] Ajayi IO, Browne EN, Garshong B, Bateganya F, Yusuf B, Agyei-Baffour P, Doamekpor L, Balyeku A, Munguti K, Cousens S, Pagnoni F (2008). Feasibility and acceptability of artemisinin-based combination therapy for the home management of malaria in four African sites. Malar J.

[32] Akweongo P, Agyei-Baffour P, Sudhakar M, Simwaka BN, Konate AT, Adongo PB, Browne EN, Tegegn A, Ali D, Traore A, Amuyunzu-Nyamongo M, Pagnoni F, Barnish G (2011). Feasibility and acceptability of ACT for the community case management of malaria in urban settings in five African sites. Malar J.

[33] Chanda P, Hamainza B, Moonga HB, Chalwe V, Pagnoni F (2011). Community case management of malaria using ACT and RDT in two districts in Zambia: achieving high adherence to test results using community health workers. Malar J.

[34] Elmardi KA, Malik EM, Abdelgadir T, Ali SH, Elsyed AH, Mudather MA, Elhassan AH, Adam I (2009). Feasibility and acceptability of home-based management of malaria strategy adapted to Sudan's conditions using artemisinin-based combination therapy and rapid diagnostic test. Malar J.

[35] Franco C, Schubert J, Yameogo M, Briggs J, Kabuya W, Hitayezu F, Gaparayi P, Rwanuza A, Nyiraharerimana E, Niyitegeka F, Mugeni C, Karema C (2008). Evaluation of the Home Based Management of Malaria Strategy in Rwanda: 2008.

[36] Hawkes M, Katsuva JP, Masumbuko CK (2009). Use and limitations of malaria rapid diagnostic testing by community health workers in war-torn Democratic Republic of Congo. Malar J.

[37] Ishengoma DS, Francis F, Mmbando BP, Lusingu JP, Magistrado P, Alifrangis M, Theander TG, Bygbjerg IC, Lemnge MM (2011). Accuracy of malaria rapid diagnostic tests in community studies and their impact on treatment of malaria in an area with declining malaria burden in north-eastern Tanzania. Malar J.

[38] Kallander K, Tomson G, Nsungwa-Sabiiti J, Senyonjo Y, Pariyo G, Peterson S (2006). Community referral in home management of malaria in western Uganda: a case series study. BMC Int Health Hum Rights.

[39] Kelly JM, Osamba B, Garg RM, Hamel MJ, Lewis JJ, Rowe SY, Rowe AK, Deming MS (2001). Community health worker performance in the management of multiple childhood illnesses: Siaya District, Kenya, 1997–2001. Am J Public Health.

[40] Ngasala BE, Malmberg M, Carlsson AM, Ferreira PE, Petzold MG, Blessborn D, Bergqvist Y, Gil JP, Premji Z, Martensson A (2011). Effectiveness of artemether-lumefantrine provided by community health workers in under-five children with uncomplicated malaria in rural Tanzania: an open label prospective study. Malar J.

[41] Rwanda MOH (2009). Community Case Management: Evaluation Report of Community Health Workers' Performance.

[42] Sirima SB, Konate A, Tiono AB, Convelbo N, Cousens S, Pagnoni F (2003). Early treatment of childhood fevers with pre-packaged antimalarial drugs in the home reduces severe malaria morbidity in Burkina Faso. Trop Med Int Health.

[43] Thomson A, Khogali M, de Smet M, Reid T, Mukhtar A, Peterson S, von Schreeb J (2011). Low referral completion of rapid diagnostic test-negative patients in community-based treatment of malaria in Sierra Leone. Malar J.

[44] Mukanga D, Babirye R, Peterson S, Pariyo GW, Ojiambo G, Tibenderana JK, Nsubuga P, Kallander K (2011). Can lay community health workers be trained to use diagnostics to distinguish and treat malaria and pneumonia in children? Lessons from rural Uganda. Trop Med Int Health.

[45] Winch PJ, Bagayoko A, Diawara A, Kane M, Thiero F, Gilroy K, Daou Z, Berthe Z, Swedberg E (2003). Increases in correct administration of chloroquine in the home and referral of sick children to health facilities through a community-based intervention in Bougouni District, Mali. Trans R Soc Trop Med Hyg.

[46] Gilroy KE, Callaghan-Koru JA, Cardemil CV, Nsona H, Amouzou A, Mtimuni A, Daelmans B, Mgalula L, Bryce J, on behalf of the CCMMQoCWG (2012). Quality of sick child care delivered by health surveillance assistants in Malawi. Health Policy Plan.

[47] Ajayi IO, Browne EN, Bateganya F, Yar D, Happi C, Falade CO, Gbotosho GO, Yusuf B, Boateng S, Mugittu K, Cousens S, Nanyunja M, Pagnoni F (2008). Effectiveness of artemisinin-based combination therapy used in the context of home management of malaria: a report from three study sites in sub-Saharan Africa. Malar J.

[48] Nsabagasani X, Jesca Nsungwa S, Kallander K, Peterson S, Pariyo G, Tomson G (2007). Home-based management of fever in rural Uganda: community perceptions and provider opinions. Malar J.

[49] Counihan H, Harvey SA, Sekeseke-Chinyama M, Hamainza B, Banda R, Malambo T, Masaninga F, Bell D (2012). Community health workers use malaria rapid diagnostic tests (RDTs) safely and accurately: results of a longitudinal study in Zambia. Am J Trop Med Hyg.

[50] Hamer DH, Brooks ET, Semrau K, Pilingana P, MacLeod WB, Siazeele K, Sabin LL, Thea DM, Yeboah-Antwi K (2012). Quality and safety of integrated community case management of malaria using rapid diagnostic tests and pneumonia by community health workers. Pathogens and Global Health.

[51] Ajayi IO, Falade CO, Olley BO, Yusuf B, Gbotosho S, Iyiola T, Olaniyan O, Happi C, Munguti K, Pagnoni F (2008). A qualitative study of the feasibility and community perception on the effectiveness of artemether-lumefantrine use in the context of home management of malaria in south-west Nigeria. BMC Health Serv Res.

[52] Uzochukwu BS, Onwujekwe E, Ezuma NN, Ezeoke OP, Ajuba MO, Sibeudu FT (2011). Improving rational treatment of malaria: perceptions and influence of RDTs on prescribing behaviour of health workers in southeast Nigeria. PLoS ONE.

[53] Bisoffi Z, Sirima BS, Angheben A, Lodesani C, Gobbi F, Tinto H, Van den Ende J (2009). Rapid malaria diagnostic tests vs. clinical management of malaria in rural Burkina Faso: safety and effect on clinical decisions. A randomized trial. Trop Med Int Health.

[54] Chinkhumba J, Skarbinski J, Chilima B, Campbell C, Ewing V, San Joaquin M, Sande J, Ali D, Mathanga D (2010). Comparative field performance and adherence to test results of four malaria rapid diagnostic tests among febrile patients more than five years of age in Blantyre, Malawi. Malar J.

[55] Ansah EK, Narh-Bana S, Epokor M, Akanpigbiam S, Quartey AA, Gyapong J, Whitty CJ (2010). Rapid testing for malaria in settings where microscopy is available and peripheral clinics where only presumptive treatment is available: a randomised controlled trial in Ghana. BMJ.

[56] Kyabayinze DJ, Asiimwe C, Nakanjako D, Nabakooza J, Counihan H, Tibenderana JK (2010). Use of RDTs to improve malaria diagnosis and fever case management at primary health care facilities in Uganda. Malar J.

[57] Chandler CI, Jones C, Boniface G, Juma K, Reyburn H, Whitty CJ (2008). Guidelines and mindlines: why do clinical staff over-diagnose malaria in Tanzania? A qualitative study. Malar J.

[58] Chandler CI, Mangham L, Njei AN, Achonduh O, Mbacham WF, Wiseman V (2012). 'As a clinician, you are not managing lab results, you are managing the patient': how the enactment of malaria at health facilities in Cameroon compares with new WHO guidelines for the use of malaria tests. Soc Sci Med.

[59] Nguyen DT, Leung KK, McIntyre L, Ghali WA, Sauve R (2013). Does Integrated Management of Childhood Illness (IMCI) training improve the skills of health workers? A systematic review and meta-analysis. PLoS ONE.

[60] Kizito J, Kayendeke M, Nabirye C, Staedke SG, Chandler CI (2012). Improving access to health care for malaria in Africa: a review of literature on what attracts patients. Malar J.

[61] Colvin CJ, Smith HJ, Swartz A, Ahs JW, de Heer J, Opiyo N, Kim JC, Marraccini T, George A (2013). Understanding careseeking for child illness in sub-Saharan Africa: a systematic review and conceptual framework based on qualitative research of household recognition and response to child diarrhoea, pneumonia and malaria. Soc Sci Med.

[62] Chanda P, Hamainza B, Moonga HB, Chalwe V, Banda P, Pagnoni F (2011). Relative costs and effectiveness of treating uncomplicated malaria in two rural districts in Zambia: implications for nationwide scale-up of home-based management. Malar J.

[63] Nonvignon J, Chinbuah MA, Gyapong M, Abbey M, Awini E, Gyapong JO, Aikins M (2012). Is home management of fevers a cost-effective way of reducing under-five mortality in Africa? The case of a rural Ghanaian District. Trop Med Int Health.

[64] Agyei-Baffour P, Hansen KS, Browne EN, Magnussen P (2012). The amount and value of work time of community medicine distributors in community case management of malaria among children under five years in the Ejisu-Juaben District of Ghana. Malar J.

[65] Okwundu CI, Nagpal S, Musekiwa A, Sinclair D (2013). Home- or community-based programmes for treating malaria. Cochrane Database Syst Rev.

[66] Kalyango JN, Lindstrand A, Rutebemberwa E, Ssali S, Kadobera D, Karamagi C, Peterson S, Alfven T (2012). Increased use of community medicine distributors and rational use of drugs in children less than five years of age in Uganda caused by integrated community case management of fever [Special supplement on Integrated Community Case Management]. Am J Trop Med Hyg.

[67] Kalyango JN, Rutebemberwa E, Karamagi C, Mworozi E, Ssali S, Alfven T, Peterson S (2013). High adherence to antimalarials and antibiotics under integrated community case management of illness in children less than five years in eastern Uganda. PLoS ONE.

[68] Ndyomugyenyi R, Kabali AT (2010). Community-directed interventions for integrated delivery of a health package against major health problems in rural Uganda: perceptions on the strategy and its effectiveness. In Health.

[69] Callaghan-Koru JA, Hyder AA, George A, Gilroy KE, Nsona H, Mtimuni A, Bryce J (2012). Health workers' and managers' perceptions of the integrated community case management program for childhood illness in Malawi: the importance of expanding access to child health services. Am J Trop Med Hyg.

[70] Nsona H, Mtimuni A, Daelmans B, Callaghan-Koru JA, Gilroy K, Mgalula L, Kachule T, Zamasiya T (2012). Scaling up integrated community case management of childhood illness: update from Malawi [Special supplement on Integrated Community Case Management]. Am J Trop Med Hyg.

[71] Rowe SY, Kelly JM, Olewe MA, Kleinbaum DG, McGowan JE, McFarland DA, Rochat R, Deming MS (2007). Effect of multiple interventions on community health workers' adherence to clinical guidelines in Siaya district, Kenya. Trans R Soc Trop Med Hyg.

[72] Mukanga D, Tibenderana JK, Peterson S, Pariyo GW, Kiguli J, Waiswa P, Babirye R, Ojiambo G, Kasasa S, Pagnoni F, Kallander K (2012). Access, acceptability and utilization of community health workers using diagnostics for case management of fever in Ugandan children: a crosssectional study. Malar J.

