# Supplementary material for: Community Health Workers and Stand-Alone or Integrated Case Management of Malaria: A Systematic Literature Review
Source: Am J Trop Med Hyg. 2014 Sep 3;91(3):461–70. doi: 10.4269/ajtmh.14-0094 (PMC4155545; doi:10.4269/ajtmh.14-0094)
Supplement: Supplementary file 1 [file SD2.pdf]

Supplemental Table 2

Overview of included studies*

Author, year of publication	Country, setting and scale	Malaria treatment and diagnosis policy	Intervention overview	Evaluation design and key outcomes	Facilitators and barriers to high quality performance of community case management
Community-based management of malaria only; presumptive diagnosis and treatment					
Ajayi and others, 2008[31,47,51]	Ghana (2 sites), Nigeria and Uganda; Rural; District	AS-AQ (Ghana); AL (Nigeria, Uganda), Presumptive	Presumptive Tx of uncomplicated malaria with ACT; inform caretakers of how to take full dose, danger signs, adverse events; referral of children with danger signs. Community meetings; handouts of Tx instructions given to caretakers.	Post-only no control. % febrile U5s seen by CHW treated with correct AM and dose: 98.0% (1,019 of 1,040) in Nigeria; 99.0% (4,473 of 4,518) and 98·0% (3,900 of 3,979) in 2 Ghana sites; 97.0% (10,671 of 11,000) in Uganda. % febrile U5s seen by CHW treated promptly and correctly: 79.9% (231 of 289) in Nigeria; 87.2% (252 of 289) and 74.2% (69 of 93) in 2 Ghana sites; 70.8% (438 of 619) in Uganda. D28 PCR adjusted cure rate: 90.9% (140 of 154) in Nigeria; 91.4% (160 of 197) in Ghana; 97.2% (210 of 216) in Uganda.	Facilitators: Overall acceptance of ACTs and intervention by caretakers, CHWs and health staff. No need for transport, lower drug costs. Easy access. Pre-packed drugs facilitated adherence to dose. HBMF well established in Uganda. Barriers: Sustainability seen as challenge (free drugs, CHW motivation, training). More incentives requested by CHWs. Negative experiences with health staff, led to bypassing of HFs in Nigeria (preferred community leader supervision, drugs directly from LGA stores).
Akweongo and others, 2011[32]	Burkina Faso and Ghana; Urban; Sub-district	AS-AQ (Burkina Faso and Ghana); AL (Burkina Faso), Presumptive	Presumptive diagnosis a nd Tx with ACT, counseling caretakers on dosage. Community mobilization not described	Post only no control. % febrile U5s seen by CHW treated with correct AM and dose: 94.8% (1,818 of 1,918) in Ghana; 93.2% (890 of 955) in Burkina Faso. % febrile U5s seen by CHW treated promptly and correctly: 18.4% (52 of 282) in Ghana; 68.7% (123 of 179) in Burkina Faso.	Facilitators: Pre-packaged drugs effective and affordable. CHWs convenient, provided quick Tx, occasional home visits. Intensive supervision (study team) encouraged good CHW performance. Barriers: Rural-style IEC ineffective and wider range of alternative Tx sources. Low community confidence in CHW training and skills. CHW unavailable. Inability to pay.
Chinbuah and others, 2006[27]	Ghana; Rural; Sub-district	AL Presumptive	Presumptive diagnosis and Tx of malaria with AL; identify danger signs for referral. Initial community meetings to explain study, discuss number of CHWs needed, nomination and acceptance of CHW. IEC on AL given to community groups for first 2 months	Pre-post no control. 20% (n = ?) sought any care < 24 hours pre-intervention, compared with 89.1% (299 of 334) post-intervention. 98.3% (354 of 360) febrile U5s seen by CHW received correct AM and dose. 29.4% (5 of 17) children with severe signs referred to HF; 60% (3 of 5) of these completed.	Facilitators: CHW confidence increased with observed patient recovery; CHW commitment and interest appreciated by community; longer training; close supervision by research team; took initiative in home follow up. Barriers: Financial motivation required for sustainability (e.g., US$20–50/month), community prefer external provision or small consultation charge (US $0.02–0.05). CHWs would rather have money than payment in kind.

(continued)

Supplemental Table 2
Continued

Author, year of publication	Country, setting and scale	Malaria treatment and diagnosis policy	Intervention overview	Evaluation design and key outcomes	Facilitators and barriers to high quality performance of community case management
Eriksen and others, 2010[15]	Tanzania; Rural; Sub-district	SP, Presumptive	Presumptive Tx of malaria with SP by women leaders; follow-up on days 2 and 3. Referral of severe malaria or non-malaria. Community sensitization meetings after selection and training of women leaders on aims of project and need to seek prompt Tx for fever from women leaders.	Cluster RCT. 97·8% (2,616 of 2,675) febrile U5s seen by women leaders received SP and paracetamol. 2.2% (59 of 2,675) referred to HF; completion rate NA. Prevalence of moderate anemia (hemoglobin level < 8 g/dL) pre- and post-intervention: 43.9–0.8% in intervention and 30.8–0.17% in control villages ($P = 0.04$).	Facilitators: In addition to intervention, increased ITN levels and food security may explain reductions in anemia in intervention and control villages. Barriers: Potential contamination between intervention and control areas; anemia may not be most appropriate outcome as non-specific for malaria intervention
Fapohunda and others, 2004[24]	Uganda; Rural; District	CQ-SP Presumptive	Presumptive Tx using pre-packaged CQ-SP (Homapak). Sensitization of communities and meetings to select CHWs. CHWs promoted prompt care seeking for malaria.	Pre-post with control. Tx with correct AM: 14.0% (134 of 959) and 37.0% (671 of 1,815) before and after in intervention districts, 3.4% (17 of 431) and 7.0% (70 of 995) in control group; overall +20% difference ($P < 0.001$)	Facilitators: None reported. Barriers: Stock-outs at HFs supporting CHWs; not all districts had achieved full implementation. Insufficient skills at HF to supervise and treat referrals. Need to improve use of CHW reports.
Franco and others, 2008[35]	Rwanda; Rural; District	AL, Presumptive	Presumptive Tx of fever with pre-packaged AL (PRIMO); RDTs and iCCM pilot just starting at time of study. Village sensitization meetings.	Post-only no control. 92.0% (370 of 402) febrile U5s seen by CHW treated with correct AL dose. 3-month review of 72 CHWs: 92 children referred (n = ?); 69.6% (64 of 92) counter-referrals completed	Facilitators: Good availability of AL in HFs and CHWs. CHWs acceptable to caretakers, recognize capacity and limitations. AL affordable. CHW have pride in serving community. Barriers: Infrequent supervision caused by other priorities, lack of time and fuel. Demand for capacity to treat more diseases. Improvements needed in counseling and checking for danger signs. Some requests for bonuses and equipment.
Kallander and others, 2006[38]	Uganda; Rural; Sub-district	CQ-SP Presumptive	Presumptive Tx using CQ-SP (Homapak); danger signs for immediate verbal referral to nearest HF; follow-up after 3 days. Community mobilization not described.	Post-only no control. Case series follow-up of referrals of 40 CDDs. Overall referral rate of 8.0% (117 of 1,454); 16 lost to follow-up, 24 excluded because of non-febrile illness. 87.0% (67 of 77) completed referral at hospital or health center.	Facilitators: High completion of referral may be caused by low costs for primary care seeking. Promptness greater for U1s or urgent referrals. Barriers: Lack of money and mother not being told to go immediately, child improved. Lack of regular follow-up by CHW. Recommend use of referral and counter-referral forms
Kidane and Morrow, 2000[16]	Ethiopia; Rural; Sub-district	CQ Presumptive	Mother co-ordinators taught other mothers to recognize malaria and treat with CQ; pictorial treatment charts of dosage; referrals; record births and deaths. Initial community sensitization meetings.	Cluster RCT. All-cause U5 mortality of 29.8 vs. 50.2 per 1,000 child-years in intervention vs. control clusters; mortality rate reduction of 40.6% (95% CI = 29.2–50.6%). 18.6% (13 of 70) potential malaria deaths in intervention vs. 56.7% (68 of 120) in control clusters.	Facilitators: Post-conflict setting means disciplined population used to self-support, strong community solidarity, no alternative income opportunities for mother coordinators

(continued)

Supplemental Table 2
Continued

Author, year of publication	Country, setting and scale	Malaria treatment and diagnosis policy	Intervention overview	Evaluation design and key outcomes	Facilitators and barriers to high quality performance of community case management
Kouyate and others, 2008[17]	Burkina Faso; Rural; Sub-district	CQ Presumptive	Women leaders led educational meetings with other mothers; held pre-packaged CQ and paracetamol stocks for presumptive Tx of febrile children; home visits; referral of severe illness. Community sensitization campaign after selection and training of women leaders.	Cluster RCT. Prevalence of moderate anemia (< 24% hematocrit) before and after: 28.0% to 16.7% in intervention and 29.9% to 14.5% in control villages ($P = 0.32$). Tx of fever with CQ before and after: 36.9–86.3% ($P = 0.002$) in intervention and 65.9–67.7% in control villages ($P = 0.71$); overall +47.6% difference ($P < 0.001$)	Facilitators: Strong experience of community support, most villages have women's agricultural groups; intervention increased coverage of appropriate malaria Tx (but not health impact). Barriers: Potential contamination between intervention and control villages; higher malaria Tx control villages at baseline; increasing CQ resistance.
Nsabagasani and others, 2007; Nsungwa- Sabiiti and others, 2007[26,48]	Uganda; Rural; National	CQ-SP Presumptive	Free distribution of pre-packaged CQ-SP (Homapak) to all U5s with fever; counseling during consultations and village meetings using pictorial charts. CHWs mobilized communities to seek prompt care for fever through village meetings and consultations.	Pre-post with control. Prompt and effective Tx: 7.4% (12 of 163) and 13.5% (21 of 156) before and after in intervention group, 7.3% (7 of 96) and 0% in control group; overall +13.5% difference ($P = 0.01$). 32.3% (13 of 39) treated with Homapak received full dose < 24 hours; 1.3% (1 of 72) treated with other AM (OR = 37.0, 95% CI = 4.8–286; $P = 0.001$). 23% Homapak use by febrile children in poorest wealth quintile, 50% in least poor (n = ?).	Facilitators: High appreciation of Homapak as accessible, free and effective in reducing severe malaria; Pre-packaging helped improved adherence to dose and duration. Barriers: Insufficient community sensitization or ownership of HBMF caused by rapid scale-up; demand for integrated management of diseases; CHW and communities concerned about lack of financial and supervisory support from HWs for motivation; reasons for SES inequities unclear.
Sirima and others, 2003[42]	Burkina Faso; Rural; Regional	CQ Presumptive	CHWs promote recognition of malaria symptoms, provide advice on Tx and referral; sell caretakers pre-packaged CQ; emphasis for malaria Tx on caretakers. Sensitization of communities, involving treatment charts, posters, radio jingles.	Post-only no control. 5·1% (93 of 1,806) children promptly receiving pre-packaged CQ progressed to severe malaria, 11.0% for those (153 of 1,396) that did not adjusted OR = 0.47 (95% CI = 0.35–0.64, $P < 0.001$). 56.4% (1,806 of 3,202) children with fever treated promptly with pre-packaged CQ from CHW.	Facilitators: Higher ratio CHW: population increased proportion children promptly accessing pre-packaged CQ from CHW. Lower cost of pre-packaged AMs. Barriers: Still some problems with incorrect dose suggest further improvements in pre-packaging needed e.g. blister-packs
Staedke and others, 2009[19]	Uganda; Urban; Sub-district	AL; CQ-SP for HBMF Presumptive	Intervention arm: mothers given pre-packaged AL, stocks replenished during monthly visits by study team. Control arm: usual Tx practice. Community mobilization not described.	Cluster RCT. 51.5% (444 of 862) febrile illnesses treated effectively < 24 hours in intervention arm, 5.2% (30 of 570) in standard care arm. Post-intervention parasite prevalence 2.0% (4 of 200) in intervention arm and 10.0% (17 of 170; $P = 0.006$) in standard care arm. Median provider costs per child per year: US$33.83 (intervention), US$23.16 (control) median user costs: US$6.00 (intervention), US$8.74 (control).	Facilitators: Having AMs in home improved prompt Tx of fever, but likely to have resulted in considerable over treatment in this urban setting. Barriers: Higher cost and few benefits when compared with standard care, authors conclude this approach may not be appropriate for an urban setting such as Kampala

(continued)

Supplemental Table 2
Continued

Author, year of publication	Country, setting and scale	Malaria treatment and diagnosis policy	Intervention overview	Evaluation design and key outcomes	Facilitators and barriers to high quality performance of community case management
Community-based management of malaria only; confirmed diagnosis with RDT and treatment					
Akweongo and others, 2011[32]	Ethiopia and Ghana; Urban; Sub-district	AL (Ethiopia); AS-AQ (Ghana), Confirmed (RDT)	Tx of RDT-positive children with ACT; counseling caretakers on dosage. Community mobilization not described.	Post only no control. % children with positive RDT result treated with correct ACT dose: 99.6% (1,206 of 1,211) in Ethiopia; 97.7% (888 of 909) in Ghana. % children with confirmed malaria treated promptly and correctly: 56.3% (18 of 32) in Ethiopia; 34.9% (37 of 106) in Ghana.	Facilitators: Pre-packaged drugs effective and affordable. CHWs convenient, provided quick Tx, occasional home visits. Intensive supervision (study team) encouraged good CHW performance. Barriers: Rural-style IEC ineffective and wider range of alternative Tx sources. Low community confidence in CHW training and skills. CHW unavailable. Inability to pay.
Chanda and others, 2011[33,62]	Zambia; Rural; Sub-district	AL Confirmed (RDT)	Tx of RDT-positive children with AL; referral of RDT-negative children with suspected non-malaria illness to nearest HF. Community mobilization not described.	Post-only no control. % children with positive RDT result treated with correct ACT dose: 99.3% (2,282 of 2,298); % children with negative RDT result treated with ACT: 0.2% (13 of 7,549). 40.2% (328 of 815) of CHW referrals completed. Cost per case (2009 US$) appropriately diagnosed and treated: 4.22 for CCM, 6.61 for health facility; ICER 4.18 (4.22–6.1)/(1.0–0.3). Provider perspective only.	Facilitators: Continuous availability of RDT; regular supervision; useful job aids. Increased accessibility and availability of CHWs vs. HFs. Barriers: Incomplete written referral feedback by HWs for CHWs; need improved communication. 3 CHWs supervised at HF, dislike caused by cost of transport. Some patient demands for AM with RDT-negative results; demand to treat non-malaria illness (iCCM)
Counihan and others, 2012[49]	Zambia; Rural; District	AL; Presumptive	Half-day training plus job aid for existing CHWs on RDT use; treatment of RDT-positive patients with AL. Community mobilization not described.	Post-only no control. Mean of 88% (n = 63) 8 critical steps for RDT use completed correctly 3 months post-training, 100% at 6 months (n = 61) and 12 months (n = 59). Positive RDT results read correctly: 97% (n = 63), 98% (n = 61), and 95% (n = 59), respectively.	Facilitators: Time since training and younger CHWs more likely to complete all critical RDT steps. Barriers: Difficulty obtaining RDTs after initial stock. Decreasing ability to interpret weakly-positive test results at 12 months. Difficulties using plastic pipette. Some facility managers resistant to CHWs using RDTs. No supervision by DHMT.
Elmardi and others, 2009[34]	Sudan; Rural; Sub-district	AS-SP Confirmed (RDT)	Tx of RDT-positive patients with AS-SP; pre-referral rectal AS for severe malaria; VHC formed to support and supervise CHWs. Community leaders sensitized villages; CHW graduation ceremony.	Post-only no control. 14 of 20 (70.0%) CHWs treated appropriately according to RDT result, remaining 6 gave AS-SP to RDT-positive patients by using clinical judgment. 0.9% (35 of 3,889) cases referred to hospital with non-malaria febrile illness.	Facilitators: Acceptance of CHWs and caring attitude; easy, close, affordable service. Barriers: Financial motivation insufficient for 13 of 20 CHWs because of time away from usual work; most believed that they were supported by VHC Request for expansion of services CHWs can provide.

(continued)

Supplemental Table 2
Continued

Author, year of publication	Country, setting and scale	Malaria treatment and diagnosis policy	Intervention overview	Evaluation design and key outcomes	Facilitators and barriers to high quality performance of community case management
Harvey and others, 2008[29]	Zambia; Rural; Sub-district	ACT (non- specified), Presumptive	Existing CHWs divided in to 3 arms: RDT manufacturer's instructions only, job aid-only, job aid plus training. Performance assessed in local HC with real patients, though all treated presumptively as per current policy. Community mobilization not described.	Post-only with control. Mean of 57% (n = 32) 16 steps for RDT use completed correctly in manufacturer's guidelines arm, 80% (n = 21) in job aid-only arm ($P < 0.05$), 90% (n = 26) job aid and training arm ($P < 0.05$). RDT results read correctly: 54% (n = 32), 82% (n = 21, $P < 0.05$) and 93% (n = 26, $P < 0.05$), respectively. Training cost US$175 per CHW.	Facilitators: Interactive training with demonstration and practice; sufficient ratio of trainers to CHWs to observe and counsel on performance. Practice improved performance in all 3 arms, although not as much as job aid or training. Barriers: Common errors: obtaining sufficient blood for RDT, waiting 15 minutes for result, interpreting results (especially weakly-positive results or invalid tests).
Hawkes and others, 2009[36]	Democratic Republic of the Congo; Rural; sub-district	AS-AQ Presumptive	Existing CHWs trained on RDTs. Performance assessed in village with real patients, though all treated presumptively as per current policy. Community mobilization not described.	Post-only no control. Median of 100% (n = 12) of 16 steps for RDT use completed correctly; 100% CHWs read RDT results correctly. At 80% prevalence, decision model found cost of $8.79 per unnecessary course of ACT averted; ~8 times cost of ACT dose, or 60% annual per capita health budget	Facilitators: High acceptance of RDTs by CHWs (utility and ease of use); experienced CHWs therefore quickly picked up new RDT skills. Barriers: At very high levels of parasite prevalence, RDTs not cost effective; associated costs likely to be major barrier to integration of RDT use for CHW
Ishengoma and others, 2011[37]	Tanzania; Rural; Sub-district	SP then AL, Presumptive then confirmed	Tx with SP changed to AL in Jan 2007 and presumptive diagnosis to confirmed (only for persons >5 years of age); referral of severe cases. Villages informed to consult CHWs for febrile illness at any time of day or night.	Post-only no control. Pre-RDTs, 98.6% (5,478 of 5,556) patients treated with SP. Post-RDTs, 96.6% (4,122 of 4,267) U5s treated with AL; 88.8% (3,942 of 4,440) persons > 5 years of age treated were RDT positive, 10.7% (475 of 4,440) were RDT positive.	Facilitators: Intensive support supervision (weekly) by experienced team; level of training. Barriers: Supervision too intense for routine implementation; will need to design sufficient program to maintain CHWs' confidence and guideline adherence.
Lemma and others, 2010[25]	Ethiopia; Rural and urban; District	AL for *P. falciparum*; CQ for *P. vivax*. Presumptive then confirmed	Year 1: CHWs in int. district treated all fever as *P. falciparum* malaria; Year 2: Half in CHWs given RDTs, RDT-positive patients treated with AL, RDT-negative patients assumed to be infected with *P. vivax* and treated with CQ. Control: malaria prevention only. Community mobilization not described.	Pre-post with control. Significant difference in malaria-specific mortality between intervention and control clusters (adjusted IRR = 0.60; 95% CI = 0.40–0.90, $P = 0.01$), no significant difference in all-cause mortality (IRR = 1.03; 95% CI:= 0.87–1.21, $P = 0.75$)	Facilitators: Gametocidal effect of AL may have reduced malaria transmission in intervention district (3-fold lower burden); similar levels of vector control in both districts. Barriers: No process data on CHW practice to determine if higher % malaria cases treated appropriately; malaria deaths identified using VA model; sampling for VA unclear.

(continued)

Supplemental Table 2
Continued

Author, year of publication	Country, setting and scale	Malaria treatment and diagnosis policy	Intervention overview	Evaluation design and key outcomes	Facilitators and barriers to high quality performance of community case management
Mubi and others, 2011[18]	Tanzania; Rural; Sub-district	AL, Presumptive	Comparison of presumptive vs. confirmed diagnosis of malaria; Tx with AL and paracetamol for all febrile patients; recognition of danger signs for referral; follow-up on days 3 and 7. Community sensitization meetings.	RCT. % children with positive RDT result treated with correct ACT dose: 99.3% (731 of 733); % children with negative RDT result treated with ACT: 6.1% (44 of 724); 96.5% (1,422 of 1,473) febrile patients in presumptive weeks given ACT. 17.2% (250 of 1,457) referral rate in RDT group compared with 5.0% (73 of 1,473) in presumptive group	Facilitators: High CHW compliance with guidelines; CHWs encourage compliance among patients, along with pre-packaged doses and monitoring completion
Ngasala and others, 2011[40]	Tanzania; Rural; Sub-district	AL Confirmed (RDT)	Treatment of RDT-positive patients with AL; referral of non-malaria fever and severe cases. Community mobilization not described.	Post-only no control. 95.1% PCR adjusted cure rate at day 28 (229 of 241); 93.0% at day 42 (224 of 241)	Not described
Thiam and others, 2012[30]	Senegal; Rural; National	AS-AQ; Confirmed (RDT)	Treatment of RDT-positive patients with AS-AQ; referral of RDT-negative patients, severe cases, pregnant women, infants < 2 months of age. Sensitization meetings, community mobilization events, and radio spots, support of head of village.	Post-only with control. % children with positive RDT result treated with correct ACT dose: 96.6% (4,126 of 4,270). 54.6% (6,871 of 12,582) patients referred; most RDT positive (94·4%, 6,486 of 6,871). Malaria mortality decreased by 62.5% (95% CI = –43.8 to –81.2%) and 23.4% (95% CI = –61.4 to 14.5%) in intervention and control areas.	Facilitators: Strong community and stakeholder involvement. Support of head of village provided important link between community and HF. Non-financial incentives.
Thomson and others, 2011[43]	Sierra Leone; Rural; Sub-district	AS-AQ Confirmed (RDT)	Treatment of RDT-positive patients with AS-AQ and paracetamol; recognition of danger signs and referral of RDT-negative patients to nearest HF. Community mobilization not described.	Post-only no control. Overall referral rate of 17.2% (2,093 of 12,204) of U5s and 8.5% (419 of 4,901) of PW. 99.3% (2,442 of 2,459) referrals caused by RDT-negative result; 0·9% (21) completed. 0.7% (17 of 2,459) referrals RDT positive with severe signs; 88.2% (15) completed	Facilitators: CHW behavior correct, need to improve referral completion, or give CHWs capacity to treat uncomplicated RDT-negative patients. Barriers: Supervision a challenge (insufficient staff). Cluster variation, perhaps caused by distance, CHW, supervision.
Community-level malaria management integrated with other health services					
Akweongo and others, 2011[32]	Malawi; Urban; Sub-district	AL Presumptive	Presumptive treatment with ACT; counseling caretakers on dosage. Plus: vaccinations, growth monitoring, nutrition, water and sanitation, family planning. Community mobilization not described.	Post-only no control. Febrile U5s seen by CHW treated with correct ACT dose: 100% (2,131 of 2,131). Febrile U5s seen by CHW treated promptly and correctly: 73.7% (590 of 801).	Facilitators: Pre-packaged drugs effective and affordable. CHWs convenient, provided quick Tx, occasional home visits. Intensive supervision (study team). Barriers: Rural-style IEC ineffective; wider range of established alternative Tx sources. Low community confidence in CHW skills. CHW unavailable. Inability to pay.

(continued)

Supplemental Table 2
Continued

Author, year of publication	Country, setting and scale	Malaria treatment and diagnosis policy	Intervention overview	Evaluation design and key outcomes	Facilitators and barriers to high quality performance of community case management
CDI Study Group, 2008; Ndyomugyen-yi and others, 2010[28,68]	Cameroon, Nigeria and Uganda; Rural; District	AL (Cameroon); ACT (Nigeria); CQ-SP (Uganda), Presumptive	Community directed intervention (CDI); presumptive treatment of malaria. Plus: different CDI tasks added in intervention districts each year (Vitamin A, TB-DOTS, ITNs for U5s and PW). Community members collectively decided health interventions, identified resources; planned how, when, where, by whom intervention will be implemented. District staff facilitated process: training, supervision, supplies.	Post-only with control. % febrile children receiving prompt and effective Tx in Cameroon: 10.5% (2 of 19) in comparison districts, 17.3% (13 of 75) after 12-month intervention (P = 0.30), 27.9% (19 of 68) after 24 months (P = 0·01). In Nigeria: 30.3% (59 of 195) in comparison districts, 62.5% (218 of 349) after 12-month intervention (P < 0.002), 77.2% (260 of 337) after 24 months (P < 0·002). In Uganda: 29.4% (5 of 17) in comparison districts, 58.5% (31 of 53) after 12-month intervention (P = 0.04), 76.7% (23 of 30) after 24 months (P = 0.002). Mean implementation costs lower in intervention than comparison districts. Salaries main cost in all sites.	Facilitators: Stakeholder consultation and mobilization; little extra advocacy needed to gain support for CDI HMM. Maturation over time. Community selection of implementers. Strong non-financial/intrinsic motivation. Community and female empowerment. HW more engaged in outreach activities. Barriers: Ensuring consistent commodity supplies (big problem in year 1); Resistance by HWs to use of CDI for TB-DOTS (too complex) and Vitamin A (HWs lose per diems from vaccination days)
Chinbuah and others, 2012; Nonvignon and others, 2012[21,63]	Ghana; Rural; District	AS-AQ, Presumptive	Presumptive treatment of fever with AL; counseling caretakers on dosage and prevention; referral of severe cases. Plus: CHWs in intervention arm trained to presumptively treat fever with AL and AMX (regardless of presence/absence of other pneumonia signs).	Cluster RCT (step-wedge). Reduction in adjusted all-cause mortality compared with standard care: 30% in AS-AQ arm (RR = 0.70, 95% CI = 0.53–0.92, P = 0.01); 44% in AS-AQ + AMX arm (RR = 0.56, 95% CI = 0.41–0.76, P < 0·001). Difference between intervention arms not significant (RR = 0.79, 95% CI = 0·56–1·12, P = 0.20). Both interventions meet WHO cost effectiveness threshold	Facilitators: Caregiver compliance high because of community sensitization and pre-packaged drugs. Treatment of malaria and pneumonia presumptive; therefore, no challenges for CHWs with respiratory rate measurement.
Gilroy and others, 2012; Callaghan-Koru and others, 2012; Nsona and others, 2012[46,69,70]	Malawi; Rural; National	AL, Presumptive	Presumptive treatment of fever with AL; referral of severe cases. Plus: assessment of pneumonia (cough with fast breathing) and diarrhea and treatment with AMX or ORS. Community orientation, village health committee monitors medicine box.	Post-only no control. % correctly assessed and classified: 92.5% (223 of 241) with uncomplicated malaria; 51.7% (30 of 58) with uncomplicated pneumonia; 90.3% (84 of 93) with uncomplicated diarrhea. % correctly treated: 79.3% (191 of 241) with malaria; 51.7% (30 of 58) with pneumonia; 68.8% (64 of 93) with diarrhea. 55.1% (38 of 69) of children with danger signs referred.	Facilitators: Strong MOH support. HSA cadre stable and well-respected in communities as salaried and formally recognized by MOH; good motivation. Strong community engagement. Barriers: Frequent stock-outs (main reason for incorrect malaria and diarrhea Tx). Problems measuring respiratory rates and classifying pneumonia. Supervision visits less frequent than planned. Skills at HFs also need strengthening.

(continued)

Supplemental Table 2
Continued

Author, year of publication	Country, setting and scale	Malaria treatment and diagnosis policy	Intervention overview	Evaluation design and key outcomes	Facilitators and barriers to high quality performance of community case management
Kalyango and others, 2012[22,66,67]	Uganda; Rural and urban; District	AL, Presumptive	Presumptive treatment of fever with AL; counseling caretakers on dosage and prevention; referral of severe cases. Plus: CHWs in intervention arm also trained to diagnose uncomplicated pneumonia using respiratory rate and treat with AMX.	Cluster RCT. % febrile children receiving correct AL dose: 100% (68 of 68) in control arm (AL only), 98.5% (56 of 57) in intervention arm (AL and AMX). % children with fast breathing receiving AMX in intervention arm: 81.7% (47 of 57). Poorest quintiles more likely to seek care from CMD than least poor (OR = 1.92; 95% CI = 0.99–3.71)	Facilitators: Training reinforced at monthly meetings with supervisors and health workers and use of job aids. High existing malaria knowledge. Community involvement and appreciation. Barriers: CHWs reported difficulty assessing respiratory symptoms and treating patients close to dose cut-off ages. Irregular supply of drugs. No reinforcement of messages after initial community sensitization.
Kelly and others, 2001; Rowe and others, 2007[39,71]	Kenya; Rural; District	SP, Presumptive	Modified IMCI algorithm to diagnose malaria; referral of severely ill children; counsel caregivers on correct Tx and preventive behaviors. Plus: integrated management of ARIs and diarrhea (clinical diagnosis only). Community mobilization not described.	Post-only no control Tx of uncomplicated malaria with SP: 41.0% (39 of 95), 92.7% (115 of 124), and 90.5% (237 of 262) after 12, 24, and 48 months. Tx of uncomplicated pneumonia with cotrimoxazole: 58.3% (28 of 48), 65.1% (43 of 66), and 50.0% (46 of 92) after 12, 24, and 48months. Referral rate for severe classifications: 70.0% (63 of 90), 42.1% (32 of 76), and 52.7% (64 of 122) after 12, 24, and 48 months.	Facilitators: Older children; no danger signs; use of job aid; CHWs perceived benefits from role (money, respect, helping community, help with chores); higher CHW education. Barriers: Long and complex guidelines, algorithm changed twice during study with remaining inconsistencies, confusing to CHWs; supervision rare; performance of supervisors no better than CHWs; worry about unnecessary referral; lack of confidence.
Mukanga and others, 2012[23]	Burkina Faso; Rural: Sub-district	AL, Confirmed	Treatment of RDT-positive patients with AL. Plus: use of respiratory rate timers to diagnose pneumonia and treat with CMX. Community mobilization not described.	Cluster RCT. 99.2% (388 of 391) RDT-positive and 3.7% (5 of 134) RDT-negative children received an ACT. 79.0% (181 of 229) with high and 38.5% (114 of 296) with normal respiratory rates received CMX. Fever clearance at day 3: 97.8% (496 of 507) in intervention and 96.3% (542 of 563) in control clusters.	Not described
Mukanga et al, 2012[23]	Ghana; Rural; Sub-district	AS-AQ, Confirmed	Treatment of RDT-positive patients with AS-AQ. Plus: use of respiratory rate timers to diagnose pneumonia and treat with AMX. Community mobilization not described.	Cluster RCT. 100% (492 of 492) RDT-positive and 3.3% (3 of 92) RDT-negative children received an ACT. 72.5% (103 of 142) with high and 44.6% (197 of 442) with normal respiratory rates received AMX. Fever clearance at day 3: 98.3% (568 of 578) in intervention and 98.6% (583 of 591) in control clusters.	Not described

(continued)

Supplemental Table 2
Continued

Author, year of publication	Country, setting and scale	Malaria treatment and diagnosis policy	Intervention overview	Evaluation design and key outcomes	Facilitators and barriers to high quality performance of community case management
Mukanga and others, 2012 and 2011[23,44,72]	Uganda; Rural; Sub-district	AL, Presumptive	Treatment of RDT-positive patients with AL. Plus: use of respiratory rate timers to diagnose pneumonia and treat with AMX. Community mobilization not described.	Cluster RCT. 99.9% (856 of 857) RDT-positive and 7.6% (9 of 118) RDT-negative children received an ACT. 98.3% (520 of 529) with high and 0.9% (4 of 446) with normal respiratory rates received AMX. Mean 96·3% score for RDT preparation; 96·0% for use of respiratory timer (14 CHW). Fever clearance at day 3: 97.6% (935 of 958) in intervention and 96.1% (922 of 959) in control clusters.	Facilitators: Lots of practice with RDTs during training led to high levels of correct interpretation. Intense supervision encouraged rational antibiotic use. Close proximity of CHWs increased community access to Tx. Barriers: Some CHW had difficulty linking diagnostic results to classification, especially pneumonia; main reason for incorrect Tx immediately post-training.
Rwanda MOH, 2009[41]	Rwanda; Rural; District	AL, Presumptive	Expanded community-IMCI tasks added to CHWs giving presumptive Tx for malaria. Plus: integrated management of pneumonia and diarrhea (clinical diagnosis only). Community mobilization not described.	Post-only no control. 84.8% (774 of 913) febrile U5s seen by CHW given correct drug and dose, 72.1% (155 of 215) with diarrhea and 79.0% (158 of 200) with pneumonia. Referral rate 18.8% (249 of 1,326); low return of counter-referrals (20–67%, numbers not detailed)	Facilitators: Caretakers satisfied with CHWs as available, welcoming, Tx received. Integration not at expense of existing malaria Tx. Barriers: Delay in supplies after initial training. Stock-outs of basic medicines caused by poor motivation of district pharmacies. Inconsistent training standards between HFs. Lack of supervision. Poor recognition of danger signs and referral.
Winch and others, 2003[45]	Mali; Rural; Sub-district	CQ, Presumptive	Additional training for CHWs on CQ doses; pictorial visual aids, recognition of danger signs for referral; training HWs on referral from CHWs. Plus: drug kits have paracetamol, eye ointment, ORS, bandages. Village sensitization meetings.	Cluster RCT. 58.9% (89 of 151) CHWs in intervention zones s old CQ at correct dose, 48.1% (63 of 131) in control zones ($P = 0.04$). HH survey: referral rate 42.1% (64 of 152) in intervention zones, 64.5% (40 of 62) completion; 11.2% (15 of 134) and 85.7% (12 of 134) in control zones. From registers: overall referral rate 16.1% (46 of 286), completion 87.0% (40 of 46).	Facilitators: Good completion rate of referrals; discrepancy between CHW records and HH survey may be caused by only recording referrals when know high chance of completion. Barriers: Compliance with correct dose lower for syrup than tablets; cost still a potential barrier as malaria season coincides with time of lowest income

(continued)

Supplemental Table 2
Continued

Author, year of publication	Country, setting and scale	Malaria treatment and diagnosis policy	Intervention overview	Evaluation design and key outcomes	Facilitators and barriers to high quality performance of community case management
Yeboah-Antwi and others, 2010; Hamer and others, 2012[20,50]	Zambia; Rural; Sub-district	AL; SP for CCM, Confirmed (RDT)	Treatment of RDT-positive patients with AL and paracetamol in intervention group, presumptively in control group. Plus: diagnosis of uncomplicated pneumonia using respiratory rate timer; Tx with AMX in intervention group, referral to nearest HF in control. Community mobilization not described.	Cluster RCT. 98.5% (267 of 271) RDT-positive and 0.4% (3 of 704) RDT-negative in intervention arm received AL. Overall, 27.5% (268 of 975) children with fever in intervention arm received AL, 99.1% (2,066 of 2,085) in control arm. 98.1% (371 of 378) with fast and 1.4% (9 of 627) with normal breathing in intervention arm received AMX. Overall, 68.2% (247 of 362) children with non-severe pneumonia received prompt and appropriate Tx in intervention group, 13.3% (27 of 203) in control group (RR = 5.32; 95% CI: = 2.19–8.94).	Facilitators: High adherence to Tx algorithms in both arms. Prompt and appropriate Tx outcomes improved with use of RDT and AMX at community level. Refresher training, frequent supervision and high education levels, simple guidelines and job aid. Good stock management. Community service main motivation. Barriers: Not seeking care from a CHW because of unavailability, severity of illness, and proximity to HF.

*ACT = artemisinin combination therapy; AL = artemether-lumefantrine; AMX = amoxicillin; AQ = amodiaquine; ARI = acute respiratory infection; AS = artesunate; CCM = community case management; CDD = community drug distributor; CDI = community-directed intervention; CHW = community health worker; CI = confidence interval; CMX = cotrimoxazole; CORP = community-owned resource person; CQ = chloroquine; DHMT = district health management team; DOTS, directly observed therapy, short course; HBMF = home-based management of fever; HF = health facility; HH = household; HMIS = health management information system; HMM = home-based management of malaria; iCCM = integrated community case management; ICER = incremental cost-effectiveness ratio; IEC = information, education, and communication; IMCI = integrated management of childhood illness; IRR = incidence rate ratio; ITN = insecticide-treated net; LGA = local government authority; MOH = Ministry of Health; NA = not available; OR = odds ratio; ORS = oral rehydration salts; PCR = polymerase chain reaction; PW = pregnant women; RDT = rapid diagnostic test; RR = relative risk; SES = socioeconomic status; SP = sulfadoxine-pyrimethamine; TB = tuberculosis; Tx = treatment; U5 = child less than five years of age; VA = verbal autopsy; VHC = village health committee.
